# Primary Pleural Benign Myxoid Schwannoma in an 18-Year-Old Female: A Case Report and Literature Review

**DOI:** 10.1155/2014/296961

**Published:** 2014-03-04

**Authors:** Hussam Abou Al-Shaar, Safi Qutob, Ahmed Abu-Zaid, Ayman Azzam, Tarek Amin, Shamayel Mohammed

**Affiliations:** ^1^College of Medicine, Alfaisal University, P.O. Box 50927, Riyadh 11533, Saudi Arabia; ^2^Oncology Center, King Faisal Specialist Hospital and Research Center, P.O. Box 3354, Riyadh 11211, Saudi Arabia; ^3^Department of General Surgery, Faculty of Medicine, Alexandria University, Alexandria 21526, Egypt; ^4^Department of Pathology and Laboratory Medicine, King Faisal Specialist Hospital and Research Center, P.O. Box 3354, Riyadh 11211, Saudi Arabia

## Abstract

Pleural schwannomas are exceedingly rare neoplasms of the thoracic cavity. To the best of our knowledge, less than 20 cases have been reported in the medical English literature. Herein, we report the case of primary pleural benign myxoid schwannoma in an 18-year-old female. The patient was originally referred to our tertiary care hospital for further management of right adrenal gland mass. Physical examination and all laboratory tests were normal. Contrast-enhanced computed tomography scan showed a 4.2 × 3.2 cm, heterogeneous noncalcified mass involving the right adrenal gland region. The right renal vein and inferior vena cava were intact. There was no pleural effusion, ascites, or lymphadenopathy. No pelvic masses were identified. Patient was scheduled for surgical resection. On laparotomy, the mass was not found in its radiologically expected location, and the right kidney and right adrenal gland were intact. The right-sided lower part of diaphragm was opened, and the mass was interestingly found inside the thorax attached to the pleura, and resected successfully. A final histopathological diagnosis of primary pleural benign myxoid schwannoma was established. At a postoperative 6-month followup, there was no radiological evidence of tumor recurrence. Furthermore, literature review on pleural schwannomas is also presented.

## 1. Introduction

Pleural schwannomas are exceedingly rare neoplasms of the thoracic cavity [1]. These tumors arise from the autonomic nerve fiber sheaths in the pleural surface of the lung [2]. To the best of our knowledge, less than 20 cases have been reported in the medical English literature so far. Pleural schwannomas are generally benign, asymptomatic, and slow growing lesions [2]. Pleural schwannomas occur more commonly in adults with male predominance [3]. Herein, we report the case of primary pleural benign myxoid schwannoma in an 18-year-old female. In addition, a literature review on pleural schwannomas is presented.

## 2. Case Report

An 18-year-old female patient was referred to our tertiary care hospital (King Faisal Specialist Hospital & Research Center) for further evaluation and management of right adrenal gland mass. Past medical history and past surgical history were unremarkable. On physical examination, no palpable mass was identified. All laboratory tests including complete blood count, renal, bone, hepatic, and coagulation profiles, carcinoembryonic antigen (CEA), alfa-feto protein (AFP), and CA (cancer antigen) 12-5 were normal.

**Figure 1:**
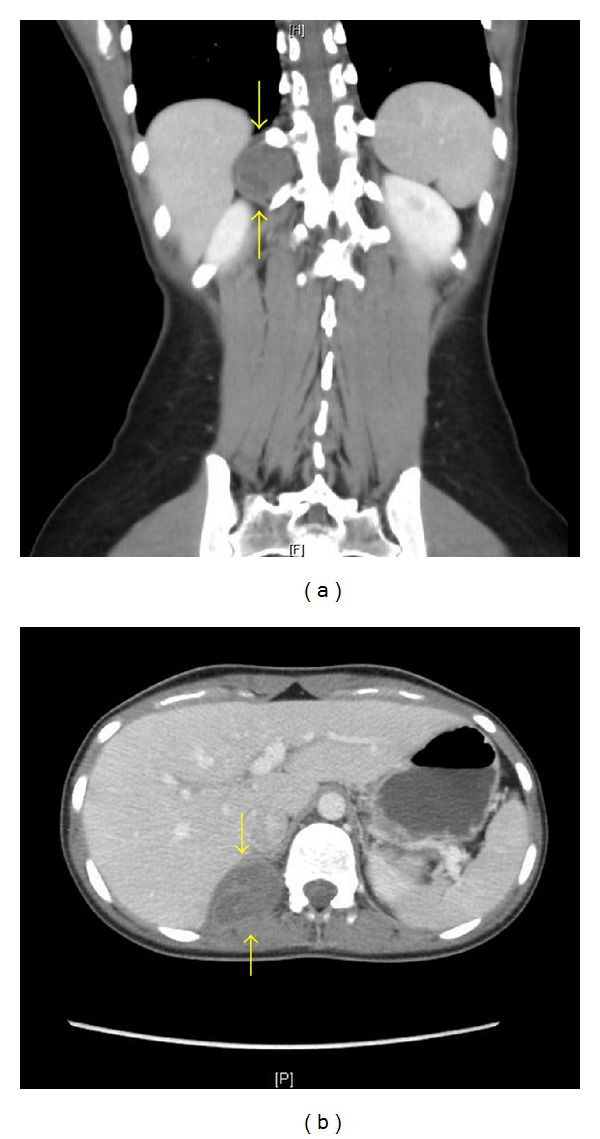
Coronal (a) and transverse (b) contrast-enhanced computed tomography (CT) scans showing a 4.2 × 3.2 cm, heterogeneous noncalcified mass involving most probably the right adrenal gland region (arrows).

Contrast-enhanced computed tomography (CT) scan showed a 4.2 × 3.2 cm, heterogeneous noncalcified mass involving most probably the right adrenal gland region (Figures 1(a) and 1(b)). The right renal vein and inferior vena cava were intact. There was no pleural effusion, ascites, or lymphadenopathy. No pelvic masses were identified. Patient was scheduled for surgical resection.

On laparotomy, the mass was not found in its radiologically expected location, and the right kidney and right adrenal gland were intact. The right-sided lower part of diaphragm was opened, and the mass was interestingly found inside the thorax attached to the pleura. The mass was resected successfully and sent for histopathological and immunohistochemical analysis.

**Figure 2:**
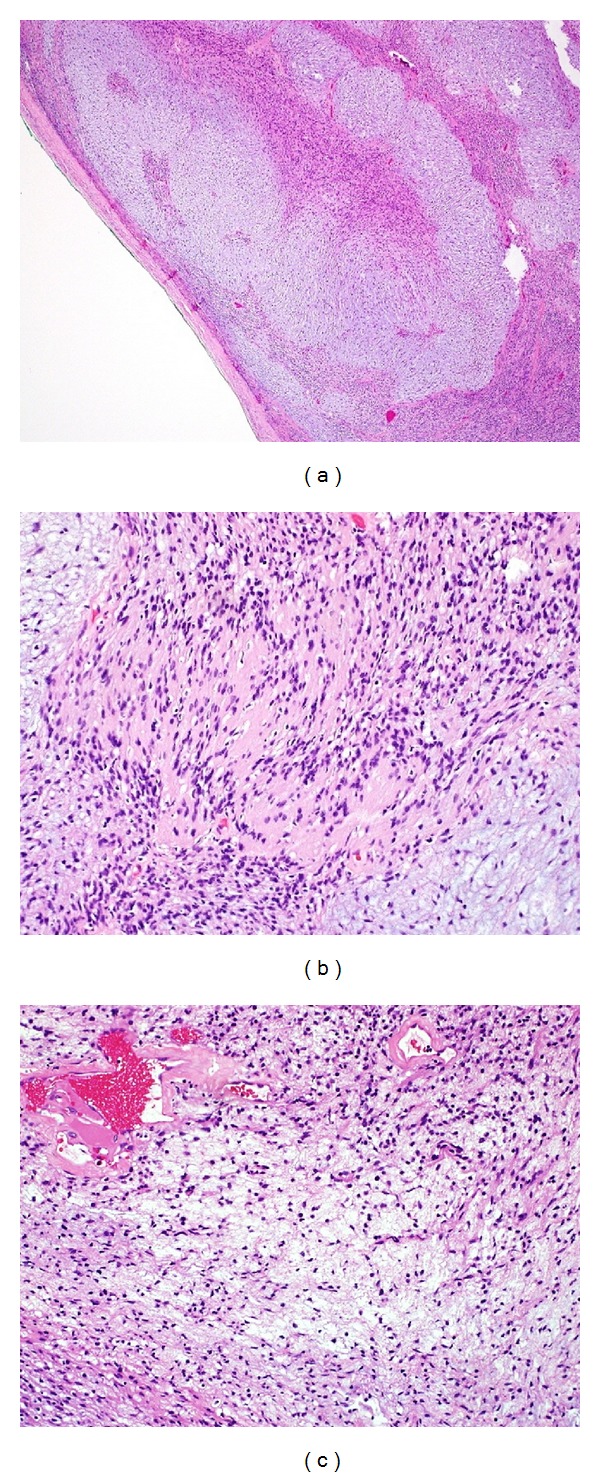
Microscopic examination of pleural mass (hematoxylin and eosin [H&E] stain). (a) Encapsulated mass with marked myxoid changes. (b) Monomorphic spindled Schwann cells forming cellular Antoni A areas with nuclear palisading and Verocay bodies alternating with hypocellular myxoid area. (c) Schwann cells with inconspicuous cytoplasm and nuclei suspended in myxoid matrix and areas of typical perivascular hyalinization.

**Figure 3:**
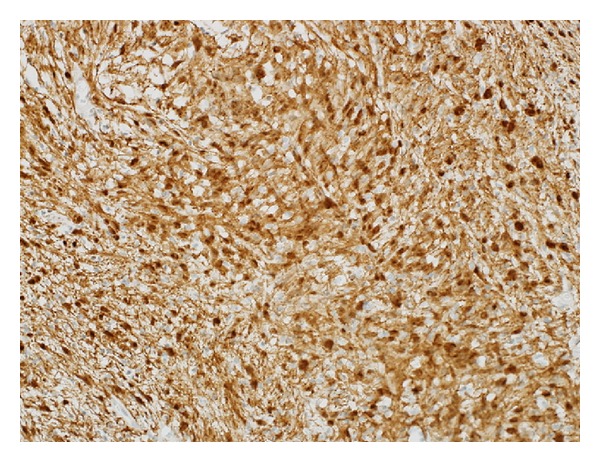
Immunohistochemical examination of the pleural mass: the tumor cells stained positive for S100 protein.

Microscopically, pathological sections from the mass showed a well-encapsulated mass with predominant myxoid areas in plexiform configuration. Alternating Antoni A cellular areas with Verocay bodies and Antoni B hypocellular areas with myxoid changes and thick-walled blood vessels with perivascular hyalinization were noted. No mitosis or necrosis was noted (Figures 2(a)–2(c)). Immunohistochemically, the tumor cells stained positive for S100 protein (Figure 3) and negative for cytokeratin, CD117, CD34, and ALK. A final histopathological diagnosis of primary pleural benign myxoid peripheral nerve sheath tumor (consistent with schwannoma) was established.

Patient had an uneventful postoperative course following surgery. Postoperatively, patient received no adjuvant therapy. At a postoperative 6-month followup, patient was completely asymptomatic and there was no radiological evidence of tumor recurrence.

## 3. Discussion

Primary pleural schwannomas are exceedingly rare neoplasms [1]. They account for 1-2% of all thoracic tumors [4]. Benign pleural schwannoma constitutes the majority of these neoplasms, yet malignancy has been reported in some cases [2, 5]. Schwannomas typically arise from specialized myelin-producing cells (Schwann cells) of either the sympathetic or parasympathetic autonomic nerve fiber sheaths [6–9]. Although it can occur at any age, pleural schwannomas commonly affect adults between their third and sixth decades. In addition, males are more commonly affected than females [3].

Schwannomas arising in the pleural surface of the lung generally grow slowly; hence, they do not usually produce symptoms. The majority of patients with pleural schwannomas are often asymptomatic or may present with nonspecific and vague symptoms. Therefore, the vast majority of pleural schwannomas are discovered incidentally during investigations for other complaints.

The majority of primary pleural schwannomas behave benignly [2]. Although they are rarely encountered, malignant pleural schwannomas have been reported in the literature [5]. Malignant lesions have been described in patients with neurofibromatosis type 1 and patients with positive history of previous radiation therapy [1, 10–12]. In addition, malignant pleural schwannomas are likely to produce symptoms due to their large size compared to benign lesions. Large tumors have the potential to produce pain and neurological symptoms due to their mass-occupying effect and compression on adjacent structures [2, 5, 13].

Diagnosing pleural schwannomas is often challenging. Definitive diagnosis cannot be reached even with advanced imaging modalities and laboratory investigations. Radiological images aid in raising the suspicion about the nature of the lesion and narrowing the differential diagnosis. Pleural schwannomas should be included in the differential diagnosis of solitary, solid, well-demarcated pleural lesions, which include, but are not restricted to, pleural lipomas, pleural metastasis, mesotheliomas, and solitary fibrous tumors. Moreover, laboratory tests usually lie within normal limits. Therefore, definitive diagnosis can only be established through histopathological examination and immunohistochemical staining of the neoplasm, which requires a section of the tumor [2].

A variety of diagnostic imaging modalities can be utilized to delineate pleural schwannomas. Computed tomography (CT) scan remains the diagnostic modality of choice for these neoplasms. CT scan can outline the size, number, and the exact location of the lesions [2]. Furthermore, CT scan can frequently demonstrate the cystic and/or solid components of tumor. Well-defined smooth round/ovoid lesions of hypodensity/isodensity with encapsulation and/or cystic degeneration are common findings on the CT scan. Contrasted CT images demonstrate heterogeneous lesions of different cellularity demarcating Antoni A and Antoni B areas. Antoni A areas typically uptake contrast and lighten up on images compared to Antoni B areas, which represent regions of cystic degeneration [2, 14, 15]. These findings aid the surgical planning of these tumors. Malignant pleural schwannomas have similar features on CT scan; however, they are usually associated with the presence of pleural nodules, pleural effusions, and metastatic pulmonary nodules [14, 16].

The vascular involvement of the tumor can be demarcated with magnetic resonance imaging (MRI), which may aid in differentiating the potential biological behavior of these lesions [9]. Pleural schwannomas are typically hyper/isointense on T1-attenuated images and heterogeneously hyperintense on T2-attenuated images [14, 15].

Microscopically, Antoni A and Antoni B areas are revealed in the majority of pleural schwannoma cases. Antoni A represents areas of hypercellularity composed of closely packed long spindle cells in palisading and interlacing fashions. In addition, Verocay bodies can also be seen in Antoni A areas. Antoni B represents areas of myxoid hypocellularity exhibiting degenerative changes (i.e., cyst formation, hemorrhage, calcification, xanthomatous infiltration, and hyalinization) [9, 17–19].

Immunohistochemically, pleural schwannomas typically stain diffusely and strongly positive for S100 protein. On the other hand, they stain negatively for CD-15, CD-30, CD-34, and pan-cytokeratin [2, 10].

The standard care of management of pleural schwannomas is primarily surgical resection (thoracoscopically or complete pleural resection) of the tumor—whenever technically possible—with frequent continuous followups [1, 2]. In all schwannoma cases, intraoperative frozen section should be carried out for histopathological examination and immunohistochemical staining in order to reach an accurate diagnosis [20].
